# Insights Into the Demands on a Clinical Oncologist in Sri Lanka

**DOI:** 10.1200/GO.21.00429

**Published:** 2022-03-01

**Authors:** Alice R.T. Bergin

**Affiliations:** ^1^ Peter MacCallum Cancer Centre, Melbourne, Victoria 3000, Australia; ^2^ Sir Peter MacCallum Department of Oncology, The University of Melbourne, Victoria 3010, Australia

Sri Lankan people with cancer have access to free, universal public health care at one of the 23 oncology treatment centers scattered across the country. Clinical oncologists working in these treatment centers are trained to provide both medical oncology and radiation services, and a private service operates in parallel.^1^ Since 1985, Sri Lanka's National Cancer Control Programme (NCCP) has been collecting cancer data of the nation's 25 districts.^2^ The NCCP hosts a user-friendly, fully searchable, and multilingual National Cancer Registry (NCR) for Sri Lanka and a separate population-based cancer registry for Colombo (PBCR—Colombo), its capital district. Recently published NCR data for 2019 indicate an overall cancer age standardized rate of 128.9 per 100,000 population.^3^ Breast, thyroid, colorectal, and gynecologic malignancies had the highest incidence among Sri Lankan women, whereas lip/tongue/mouth, trachea/bronchus/lung, colorectal, and esophageal malignancies had the highest incidence among Sri Lankan men.^3^ Sri Lankan patients with cancer are significantly underserved with an estimated one clinical oncologist per 500,000 population reported in 2019.^1^ There is little information on these important members of the cancer workforce. Workforce data are crucial as the WHO's Global Cancer Observatory estimates that by 2040, Sri Lanka's population will surpass 22 million people and new cancer cases are expected to increase by 31.4%, from 29,604 in 2020 to 38,886 new cases in 2040.^4^

In the article that accompanies this editorial, Gunasekera et al^5^ provide unique and thought-provoking insights into the clinical oncology workforce in Sri Lanka with their aptly designed, web-based, cross-sectional survey published in the *Journal of Global Oncology*. The authors set out to describe the workforce, practice setting, workload, and service provision while exploring perceived barriers to effective patient care. This survey was conducted between October 2019 and March 2020, coinciding with the onset of the crippling, global COVID-19 pandemic.

Of the 54 clinical oncologists invited to participate, 49 (76%) responded to a succinct and pertinent questionnaire (see the Data Supplement^5^). The questionnaire was designed to gather information about the respondent's past medical training, current place of work, and access to chemotherapy, radiotherapy, and palliative care services at the oncology service where they work. In addition, the authors sought specific information about the respondent's case load, on-call expectations, and access to paid leave.

The survey results are stark. More than half (64%) of the respondents are on call 7 days a week (Table 3^5^), with 79% providing both chemotherapy and radiotherapy services during the standard working day (Table 4). With a reported median 45-hour working week, 48% of respondents regularly worked more than 50 hours per week (Table 3^5^). Although the majority (81%) had access to at least one week of paid leave, nearly 60% had no access to paid academic leave (Table 3^5^). Respondents reported reviewing a median of 475 patients with a new diagnosis of cancer per year, and nearly 80% reported that these patients are seen on the day of referral, without a planned appointment, to limit the patient's commute time (Table 3^5^). With an average of 35 outpatients seen during a typical clinic working day, time is a luxurious commodity. As such, respondents reported a median 15 minutes for a new patient consultation and 7.5 minutes for a review consultation (Table 3^5^). Despite these figures, nearly 40% of respondents felt that their substantive clinical workload did not have an impact on patient care (Table 3^5^).

The most commonly reported barriers to delivery of effective patient care were complete unavailability or limited access to radiotherapy, diagnostic imaging, and/or chemotherapy services (Fig 2^5^). Other barriers reported by respondents included inadequate renumeration in the public sector; a patient's inability to pay for treatment, imaging, and/or pathology; and inadequate time for both research activities and self-directed learning. The authors noted that just 6% of respondent's time was spent doing research activities, and they postulate that the reason is high clinical workload. The authors also emphasize the scarcity of radiotherapy services in Sri Lanka as a significant barrier, highlighting that just six of the 23 oncology treatment centers offer radiotherapy services. As a result, the demands on clinical oncologists working at these six centers significantly exceed the reported median.

Since the survey period, health services across the globe have been stretched beyond capacity by the COVID-19 pandemic. At the time of writing, Sri Lanka had 580,209 new cases and 14,771 deaths from COVID-19.^6^ Sri Lanka's oncology services and workforce have been^7,8^ and will continue to be under immense pressure until the pandemic subsides. The authors call for better resourcing and optimization of current oncology services to ensure that contemporary clinical oncologists can work to their maximal potential.

Gunasekera et al^5^ have comprehensively detailed the heavy workload and pressures on a clinical oncologist working in Sri Lanka. Despite this, clinical oncologists still manage to muster the energy and compassion to see most new patients on the day of referral, thereby ensuring that the patient's first experience is a little less arduous.
